# Thermal stability of natural pigments produced by *Monascus purpureus* in submerged fermentation

**DOI:** 10.1002/fsn3.2425

**Published:** 2021-07-26

**Authors:** Fatemeh Abdollahi, Mahshid Jahadi, Mehrdad Ghavami

**Affiliations:** ^1^ Department of Food Science and Technology, Science and Research Branch Islamic Azad University Tehran Iran; ^2^ Department of Food Science and Technology Faculty of Agriculture, Isfahan (Khorasgan) Branch Islamic Azad University Isfahan Iran

**Keywords:** *Monascus purpureus*, natural pigments, response surface methodology, stability

## Abstract

The major aim of the current study was to assess thermal stability of red pigments produced by *Monascus purpureus* ATCC 16362/PTCC 5303 in submerged fermentation. Natural pigments were produced by *Monascus purpureus* using stirred tank bioreactor. Stability of *Monascus purpureus* pigments was assessed under various temperature (50.2–97.8°C), salt (0%–2.5%), and pH (4.3–7.7) values. Thermal degradation constant and half‐life value of the red *Monascus purpureus* pigments were analyzed using response surface methodology followed by a first‐order kinetic reaction. Results of this study showed that pH, temperature, and salt content could affect red color stability of *Monascus purpureus*. The pigment showed various stabilities in various thermal conditions (temperature, salt, and pH). At high temperatures, degradation constant of the red pigments increased with decreasing pH, revealing that the *Monascus* red pigment was destroyed at lower pH values and salt could affect stability of the red pigments at lower temperatures.

## Graphical abstract

Thermal degradation and half‐life value of the red Monascus purpureus pigments were analyzed using response surface methodology followed by a first‐order kinetic reaction. The major aim of this study was to assess thermal stability of red pigments produced by Monascus purpureus ATCC 16362/PTCC 5303 in submerged fermentation processes. At high temperatures, degradation constant of the red pigments increased with decreasing pH, revealing that the Monascus red pigment was destroyed at lower pH values. Salt content could affect stability of the red pigments at lower temperatures.

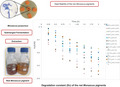

## 1 INTRODUCTION

Color is one of the most important characteristics of foods. It includes visual signals for flavor identification and taste thresholds. Furthermore, color plays significant roles in consumer satisfaction of foods (Hutchings, 2021). Legally permitted pigments are divided into two major categories of natural and synthetic. Natural colors include beneficial and therapeutic characteristics. Use of natural colors is one of the current marketing trends because of consumer's concerns about the safety of artificial food dyes, facilitated by possible health benefits of the natural pigments (Rodriguez‐Amaya, 2016). However, natural pigments show more sensitivity and instability when exposed to environmental factors such as high temperature, oxidation, and pH than artificial colors (Coultate & Blackburn, 2018; Khoo et al., 2017; Minioti et al., 2007; Wahyuningsih et al., 2017). Microbial pigments, as natural pigments (biopigments), are produced by bacteria, algae, and fungi (Aruldass et al., 2018). Fungi are further useful for the industrial production of pigments because of their fast growth rate, easy culture, ability to grow on low‐cost substrates, independent of weather conditions, production of various shades of colors, high yield of the products, and feasibility of bioprocess development. *Blakeslea trispora* and *Monascus purpureus* are examples of fungi biopigments used in food industries (Tirumale & Wani, 2018‏). In 1884, *Monascus* was first named and classified by the French Scientist, van Tieghem (Chen et al., 2015). The Genus *Monascus* belongs to the Family Monascaceae, Order Eurotiales, class Ascomycetes, Phylum Ascomycota, and Kingdom fungi. Most strains belong to three species of *M. pilosus, M. purpureus*, and *M. ruber* (Pan and Hsu, 2014). For centuries, fermented rice products have been used in Asia and Indonesia as dietary staples and food (Domínguez‐Espinosa and Webb, 2003). *Monascus* pigments (Mps) are used for controlling blood cholesterol, diabetes, and obesity and preventing cancers (Hong et al., 2008; Lee et al., 2011; Lee & Pan., 2012; Shi & Pan, 2011). These pigments also include antioxidant, antimicrobial, and antifungal characteristics. Furthermore, Mps are used as nitrite or nitrate replacements of meats (Estiasih et al., 2020; Feng et al., 2012; Nateghi et al., 2020; Seong et al., 2017). The most important pigment produced by *Monascus* sp. is red. This pigment contains monacolin K, which includes several characteristics in red koji (Lee et al., 2001).

Naturally, Mps are dissolved in water and fat (Carvalho et al., 2005), stable against pH 2–10 and high heats and usually affected by the production processes (Tseng et al., 2000). *Monascus* pigments produced by *Monascus ruber* include moderate stability when exposed at low pH or high temperatures (Silveira et al., 2013). Thermal processing is one of the most important methods of food preservation. Extreme thermal processes such as pasteurization, sterilization, and baking can affect stability of Mps (Velmurugan et al., 2011). *Monascus* pigments might tolerate pasteurization conditions. However, further studies are necessary to verify the interaction of Mps with food components on their stability (Silveira et al., 2013). Degradation constant (D_c_) is an important factor to show instability of pigments during processing (Fernández‐López et al., 2013; Silveira et al., 2013; Vendruscolo et al., 2013). Design of kinetic models for Dc of Mp under processing conditions is necessary to prepare food products. However, availability of information on reaction kinetics is quite limited and modeling of the degradation kinetics for Mps in temperature ranges of heat processing is necessary. Based on the above‐mentioned facts, the aim of the current study was to investigate effects of salt content, temperature, and pH on the stability of Mps produced by *Monascus purpureus* ATCC 16362 in submerged fermentation using response surface methodology (RSM).

## 2 MATERIALS AND METHODS

### 2.1 Preparation of fungal strains

*Monascus purpureus* ATCC 16362/PTCC 5303 was provided by the Microbial Collection Center of Iran Scientific and Industrial Research Organization, Tehran, Iran. Mycelia were cultured on yeast powder‐soluble starch (YpSS) and resuspended every 30 days in fresh culture media and incubated at 30°C for 7 days. These were stored at 4°C until use (Keivani et al., 2020).

### 2.2 Preparation of spore suspensions

To prepare spore suspensions, the fungal strain was cultured on YpSS agar media and incubated at 30°C for 7–10 days and then rinsed with 5 ml of sterile distilled water (DW). Fungal cells in suspensions were counted using light microscope and cell‐counter slides (Keivani et al., 2020).

### 2.3 Preparation and inoculation of seed media

Seed culture media were prepared with specific compounds at pH 6 and sterilized using autoclave at 121°C for 15 min. Spore suspensions were used for the inoculation of seed media and incubated (Raiman Zist Fanavar, Iran) at 30°C for 30 hr at 120 rpm (Keivani et al., 2020).

### 2.4 Preparation and inoculation of the major culture media

Using seed culture media as the final media of 10^5^ spores, inoculation was carried out in the major media optimized at 30°C for 21 days at 40 rpm (pH 4) using stirred tank bioreactor (MiniX, Yekta Tech, Iran) (Keivani et al., 2020).

### 2.5 Extraction of *Monascus* red pigments

Briefly, Mps were separated from the biomass using Whatman No. 1 filter papers. The mixing ratio of filtered materials to ethanol was 10:100. This was ultrasounded for 30 min using ultrasound device and transferred to shaking incubator for 1 hr at 180 rpm to extract red pigments (Aruldass et al., 2018).

### 2.6 Heat treatment

In general, Mps were mixed in citrate phosphate buffer to reach the selected pH values. Effects of temperature (50.2–97.8°C), salt (0%–2.5%), and pH (4.3–7.7) on Dc of the red pigments by *M*. *purpureus* ATCC 16362/PTCC 5303 were assessed using water bath. Mp solutions were heated for 2 hr using water bath (50.2–97.8°C), and samples were collected after 15‐min time intervals. Then, absorbance was measured at 500 nm using spectrophotometer (Spectronic UNICO 2100, USA) (Vendruscolo et al., 2013).

### 2.7 Kinetic calculations

The first‐order kinetic model could calculate heat Dcs. This parameter was expressed using Equation (1), and the regression lines were achieved by plotting logarithms of the remaining pigments.

(1) $$\frac{\mathrm{dA}}{\mathrm{dt}}=‐D_{c}$$

where A was the absorbance (UA_500nm_), t was the time (h), and D_C_ was the Dc (h^−1^). If boundary conditions in this formula linearized to A = A_0_ at *t* = 0 and A = A when *t* = *t*, results are shown in Equation (2).

(2) $$\text{ln}\left(\frac{\mathrm{dA}}{\mathrm{dt}}\right)=‐D_{c}.t$$

where A_0_ was the initial absorbance (UA_500 nm_). Half‐life value (*t*_1/2_) was calculated using various values of D_C_ as follows:

(3) $$t_{1/2}=\left(\frac{\text{ln}\;2}{D_{C}}\right)$$

where A/A_0_ was 2.

### 2.8 Experimental design

In the present study, temperature (A) (50.2–97.8°C), pH (B) (4.3–7.7), and salt content (C) (0%–2.5%) were analyzed as independent variables using central composite design (CCD) of RSM and Expert Design Software v.7.0.0. Levels of real variables in CCD are shown in Table 1. Effects of the significant independent variables were assessed in terms of D_C_ (Y_1_) and *t*_1/2_ (Y_2_) of the Mps using RSM at a temperature range of 50.2–97.8°C, pH range of 4.3–7.7, and salt content of 0%–2.5%. The CCD with 20 experiments (14 axial points and six replicates at the center point, *α* = 1.7) was used (Table 2). Response model was expressed using coded variables (Vendruscolo et al., 2013). The Mp solutions were heated for 2 hr using water bath (Table 2), and data were collected within 15‐min time intervals for the calculation of Dc and *t*_1/2_.

**TABLE 1:** Levels of the variables in central composite design (*α* = 1.7)

Variable	Unit	Level and range				
		−1.7	−1	0	+1	+1.7
Temperature (A)	°C	50.2	60	74	88	97.8
pH (B)	–	4.3	5	6	7	7.7
Salt (C)	%	0	0.55	1.27	2	2.5

**TABLE 2:** Degradation constants (D_C_) and half‐life value (*t*_1/2_) for various heat treatments, pH, and salt percentages of red pigments produced by *Monascus purpureus* in submerged fermentation

Experiment	Real variable			Response	
	Temperature (ºC)	pH	Salt (%)	Dc (h^−1^) (Y_1_)	*t*_1/2_ (h) (Y_2_)
1	60	5	0.55	0.104	6.64
2	88	5	0.55	0.230	3.01
3	60	7	0.55	0.098	7.07
4	88	7	0.55	0.192	3.61
5	60	5	2	0.135	5.11
6	88	5	2	0.218	3.17
7	60	7	2	0.144	4.81
8	88	7	2	0.169	4.08
9	50.2	6	1.27	0.102	6.79
10	97.8	6	1.27	0.231	3.15
11	74	4.3	1.27	0.170	4.07
12	74	7.7	1.27	0.147	4.71
13	74	6	0.04	0.145	4.75
14	74	6	2.51	0.146	4.73
15	74	6	1.27	0.135	5.12
16	74	6	1.27	0.114	6.05
17	74	6	1.27	0.103	6.67
18	74	6	1.27	0.135	5.12
19	74	6	1.27	0.114	6.05
20	74	6	1.27	0.104	6.67

## 3 RESULTS AND DISCUSSION

In this study, effects of temperature (A) (50.2–97.8°C), pH (B) (4.3–7.7), and salt content (C) (0%–2.5%) on Dc and *t*_1/2_ were investigated. Results presented in terms of A/A_0_ showed changes in Mp absorption. Since the graph was logarithmic, ln (A/A_0_) was used to linearize the graph. Based on Table 2, the highest Dc was linked to treatment 10 (97.8°C, pH 6, 1.27% salt) and the lowest Dc was associated with treatment 3 (60°C, pH 7, 0.55% salt). Regression analysis was used first for each isothermal experiment.

**FIGURE 1:**
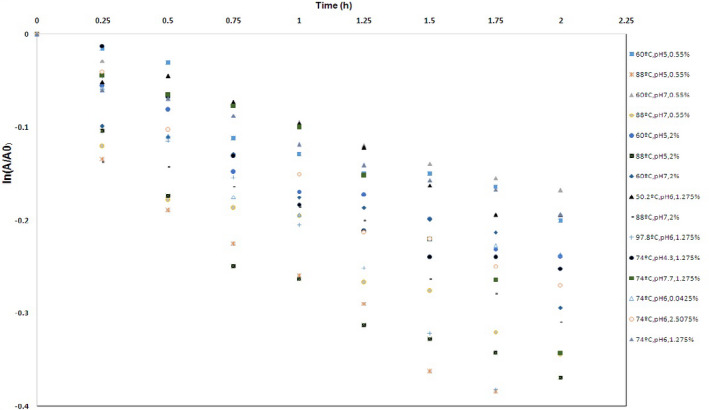
Degradation constant (Dc) of the *Monascus* pigments in aqueous solution versus various conditions [temperature (50.2–97.8°C), pH (4.3–7.7), and salt content (0%–2.5%)]

Figure 1 shows the curves of ln(A/A0) against heat treatment time. The kinetic parameters of Dc and *t*_1/2_ of the red pigments are listed in Table 2. Clearly, Mp degradation increased with increasing temperatures. Furthermore, relationships revealed that degradation of the pigments was followed by a kinetic model with a good regression coefficient of .95 ≤ *R* ^2^ ≤ .99, resembling degradation behavior of monacolin K extracted from *Monascus* sp. (Ou et al., 2009). The adjusted *R* ^2^ and predicted *R* ^2^ included .9348 and .9124, respectively. Coefficient of variation (CV) of this model included 7.85%, which showed distribution of statistical data in the samples. The RSM is reported as an effective technique for the investigation of degradation kinetics. Effects of temperature and pH linearly and interactions of temperature–pH and temperature–salt concentration were statistically significant. Furthermore, square effects of each temperature, pH, and salt percentage were significant. For a better assessment of reaction kinetics, the second‐order polynomial model was coded to all data based on the following equation:

(4) $$D_{c}=+0.12+\left(0.038A\right)‐\left(8.948E‐003B\right)+\left(3.224E‐003C\right)‐\left(0.011\text{AB}\right)‐\left(0.014\text{AC}\right)+\left(0.016A^{2}\right)+\left(0.015B^{2}\right)+\left(0.011C^{2}\right)$$

where D_C_ was the degradation constant (h^−1^), A was the variable temperature, B was the variable pH, and C was the salt percentage.

**FIGURE 2:**
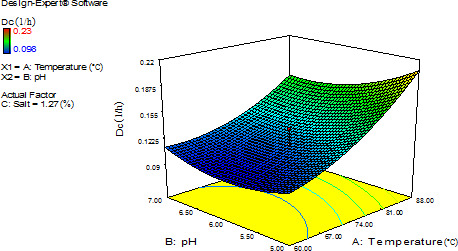
Effects of temperature and pH levels on the thermal degradation constant (Dc) of *Monascus* red pigments

As shown in Equation 4, factors of temperature and salt content were linearly linked to positive coefficient, pH was linearly linked to negative coefficient, and temperature interacted with pH and salt percentage of decreasing effects. Those with positive coefficients additively affected red pigment instability. The three‐dimensional (3‐D) curve was an upward curve and graph showed that Dc of the aqueous Mps at high temperatures (75–88°C) was more stable at pH 7 than pH 5. At low temperatures (60–75°C), Dc of the Mps was mostly stable at pH 6. Moreover, graph showed that the highest instability occurred at the highest temperature, while the most stable conditions for the Mps included pH 6 and temperature of 50°C (Figure 2). This heat‐induced pigment degradation has been reported in red and orange pigments extracted from *M. ruber* (Vendruscolo et al., 2013).

**FIGURE 3:**
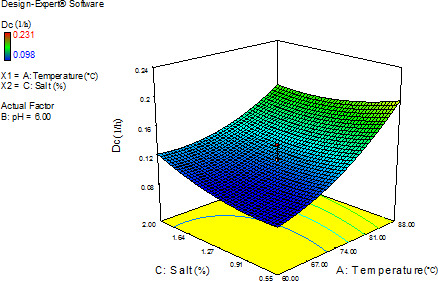
Effects of temperature and salt content on the thermal degradation constant (Dc) of *Monascus* red pigments

Stability of the red pigments of *Monascus* added to rice has studied and achieved similar results in this model. Decreased color stability due to high temperatures is expected in most natural colors, but changes in pH can include various effects on natural colors (Priatni, 2015). The 3‐D diagram showed that at 88°C, the Dc of aqueous of Mps was more stable at 0.55 compared to 2% salt, although, at 60°C, the Dc of aqueous of Mps was more stable at 2 compared to 0.55% salt (Figure 3). Furthermore, red pigments of *Monascus* produced with corn wastes can be stable in 0.5% (wt%) sodium chloride solution and 0.5% (wt.%) ammonium chloride of the media (Velmurgan et al., 2011). Fransis (1987) reported that salt‐containing solutions of red pigments of *Monascus* sp. could affect the pigment stability. Red pigments of *Monascus* sp. were more sensitive to pH, compared to that yellow and orange pigments were. Greater stability of the yellow pigments could occur due to two reasons. The first reason was that the yellow pigments were less susceptible to red pigments and its true stability and the second reason was that degradation of yellow pigments and production of yellow compounds because of the color degradation caused further persistence of the yellow pigments (Francis, 1987). Half‐life value (*t*_1/2)_ assessments showed stability of the *Monascus* red pigments under process conditions. The *t*_1/2_ of Mps was inversely linked to Dc. Half‐life value of Mps was another factor to show stability of *Monascus* red pigments. Values of *t*_1/2_ were calculated as follows:

(5) $$t_{1/2}=+5.95‐\left(1.16A\right)+\left(0.20B\right)‐\left(0.23C\right)+\left(0.55\text{AC}\right)‐\left(0.33A^{2}\right)‐\left(0.53B^{2}\right)‐\left(0.41C^{2}\right)$$

where *t*_1/2_ was the half‐life value, A was the variable temperature, B was the variable pH, and C was the salt content. As shown in Equation 5, temperature and salt content were linearly linked to negative coefficient of decrease, pH was linearly linked to positive coefficient of additive effects, and temperature interacted with salt content of additive effects. Moreover, square of the three factors was individually linked to negative coefficients, including decreasing effects on *t*_1/2_ of the Mps. The variance analysis showed significant effects of temperature, pH, and salt content on the *t*_1/2_ of Mps (*p* < .01), insignificance of lack of fit (*p* = .9325) and *R* ^2^ of .8949 (Table 3). The most effective independent variable on *t*_1/2_ was temperature. Effects of temperature linearly and square of temperature, salt, and pH on *t*_1/2_ of Mps were significant; adjusted *R* ^2^ was .8204, and predicted *R* ^2^ was .7289. The coefficient of variation in this model was 11.06%.

**TABLE 3:** Effects of temperature (A), pH (B), and salt (C) and their interactions on Dc and *t*_1/2_ of the red pigments produced by *Monascus purpureus* using RSM

Source	Sum of square Dc	*df* Dc	*F*‐value Dc	*p*‐value Dc	Sum of squares *t*_1/2_	*df* *t*_1/2_	*F*‐value *t*_1/2_	*F*‐value *t*_1/2_
Model	0.033	8	35.05	<.0001	29.12	7	14.61	<0.0001
A‐temperature	0.022	1	183.42	<.0001	18.46	1	64.8	<0.0001
B‐pH	0.001	1	9.30	.0110	0.54	1	1.9	0.1937
C‐salt	0.000	1	1.20	.2953	0.74	1	2.6	0.1329
AB	0.001	1	8.20	.0154				
AC	0.002	1	13.08	.0040	2.44	1	8.57	0.0126
A^2^	0.005	1	37.96	<.0001	1.63	1	5.72	0.034
B^2^	0.003	1	26.84	.0003	4.21	1	14.76	0.0023
C^2^	0.002	1	13.49	.0037	2.51	1	8.8	0.0118
Residual	0.001	11			3.42	12		
Lack of fit	0.000	6	0.22	.9496	0.98	7	0.29	0.9321
Pure error	0.001	5			2.43	5		
Total	0.035	19			32.54	19		

**FIGURE 4:**
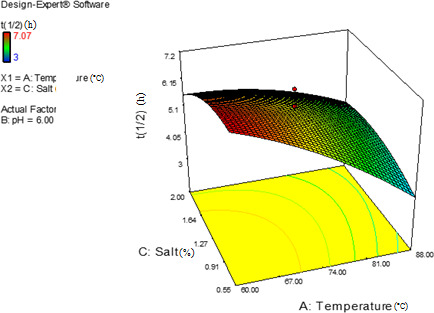
Effects of temperature and salt content on the on half‐life value (*t*_1/2)_ of *Monascus* red pigments

Figure 4 shows that the *t*_1/2_ of Mps decreased by increasing temperature and decreasing salt content; hence, the red pigment could be used in high‐salt foods such as snacks and potato chips after heat treatments. Since the half‐life of this pigment is significantly high at 74°C and includes relatively good stability in the presence of salt, the red pigment could be used in meat products such as sausages.

## 4 CONCLUSIONS

In conclusion, results of the current study have suggested stability statues of the red pigments produced by *M. purpureus* trough submerged fermentation under special conditions, in which various temperatures, salt content, and pH values resulted in pigment degradations. The kinetic model for thermal degradation of the aqueous Mps produced by *M. purpureus* in submerged fermentation was validated as the first order based on the correlations from the assessment of the degradation rate kinetic constant for each thermal processing condition. Furthermore, Mp assessments have shown that the pigments include various behaviors against various thermal processing conditions (temperature, salt, and pH). In general, Dc of Mps increased with increasing temperature but the pigment instability decreased with increasing pH at high temperatures. Moreover, results have demonstrated that the highest instability occurred at the highest temperature and the most stable conditions for the Mps included pH 6 and temperature of 50°C. Half‐life of the Mps decreased as temperature increased, while addition of salt decreased *t*_1/2_ of the aqueous Mps. Moreover, RSM analysis and empirical results have shown behaviors of D_C_ and *t*_1/2_ of the pigments produced by *M. purpureus* ATCC 16362/PTCC 5303 in submerged fermentation, revealing that kinetic equations of the color variables have represented color changes of the pigments well and, therefore, can be used to describe color degradations during heat processing and storage.

## CONFLICT OF INTEREST

The authors declare that they do not have any conflict of interest.

## ETHICAL APPROVAL

This study does not involve any human or animal testing.

## INFORMED CONSENT

Written informed consent was obtained from all study participants.

## Data Availability

The raw/processed data required to reproduce these findings cannot be shared at this time as the data also form part of an ongoing study.
